# Better Sleep at Night: How Light Influences Sleep in *Drosophila*

**DOI:** 10.3389/fphys.2020.00997

**Published:** 2020-09-04

**Authors:** Gabriella M. Mazzotta, Milena Damulewicz, Paola Cusumano

**Affiliations:** ^1^Department of Biology, University of Padova, Padua, Italy; ^2^Department of Cell Biology and Imaging, Jagiellonian University, Kraków, Poland

**Keywords:** *Drosophila*, wake-sleep pattern, light, photoreception, neurotransmitters

## Abstract

Sleep-like states have been described in *Drosophila* and the mechanisms and factors that generate and define sleep-wake profiles in this model organism are being thoroughly investigated. Sleep is controlled by both circadian and homeostatic mechanisms, and environmental factors such as light, temperature, and social stimuli are fundamental in shaping and confining sleep episodes into the correct time of the day. Among environmental cues, light seems to have a prominent function in modulating the timing of sleep during the 24 h and, in this review, we will discuss the role of light inputs in modulating the distribution of the fly sleep-wake cycles. This phenomenon is of growing interest in the modern society, where artificial light exposure during the night is a common trait, opening the possibility to study *Drosophila* as a model organism for investigating shift-work disorders.

## Introduction

**FIGURE 1:**
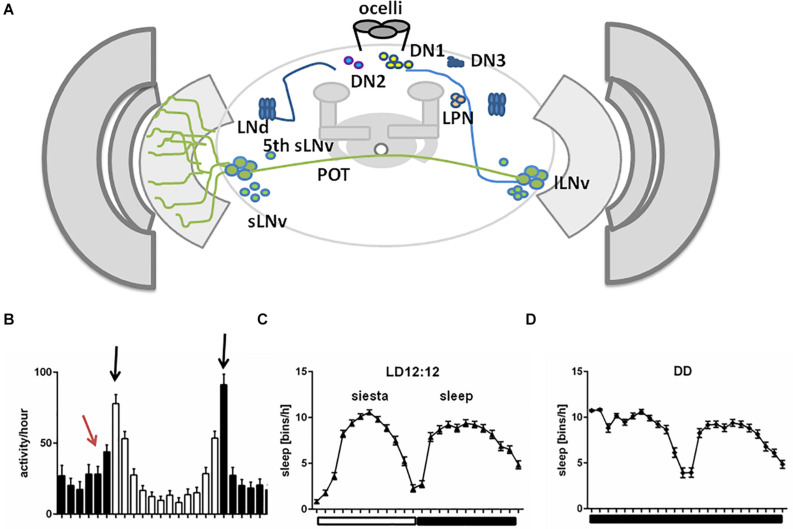
A schematized representation of clock network in *Drosophila* brain. **(A)** The main pacemaker cells, lateral ventral neurons, small (s-LNvs) and large (l-LNvs) are located in accessory medulla. l-LNvs send projections, called posterior optic tract (POT), to the contralateral hemisphere and form network of processes in the medulla neuropil, while s-LNvs innervate dorsal brain. Six lateral dorsal neurons (LNds) and three lateral posterior neurons (LPNs) are located above the main pacemakers. In the dorsal brain, three groups of dorsal neurons are located (DN1s, DN2s, DN3). **(B)** Representative activity profile in light:dark (LD12:12) conditions with pointed morning/evening peaks (black arrows) and morning anticipation (red arrow). **(C,D)** Representative sleep pattern observed in LD cycles **(C)**, with siesta during the day and sleep during the night and constant darkness (DD) conditions **(D)**.

Life on Earth has been shaped by rhythmic changes of environmental cues and living organisms have evolved endogenous mechanisms to coordinate physiological and behavioural functions. For example, in humans and other diurnal animals, most activities occur during the day, contrary to nocturnal animals, mostly active during the night. Among environmental factors, light plays a major role in adjusting temporal niches of animal behaviour in relation to natural surroundings, in the sense that it acts as an arousal signal for diurnal animals and at the same time promotes sleep in nocturnal ones (Redlin, 2001). *Drosophila* exhibits a very well-established daily activity pattern: under 12 h Light-12 h Dark cycles (LD12:12), flies display distinct morning and evening bouts of activity, separated by a prolonged siesta in the middle of the day. This behavioural output is the result of an orchestrated activity of different clusters of clock cells and signals (Grima et al., 2004; Stoleru et al., 2004; Picot et al., 2007; Cusumano et al., 2009; Zhang et al., 2009; Yao and Shafer, 2014; Chatterjee et al., 2018; Díaz et al., 2019; Schlichting et al., 2019b). In *Drosophila*, the circadian oscillator is located in about 150 neurons that, based on their anatomical location, are classified as: small and large ventral-lateral neurons (s-LNvs and l-LNvs, respectively), dorsal-lateral neurons (LNds), lateral posterior neurons (LPN), and three groups of dorsal neurons (DN1s, DN2s, and DN3s) (Schubert et al., 2018; Figure 1A). Among these, the s-LNvs and LNds are specifically involved in the control of morning and evening activity, respectively (Grima et al., 2004; Stoleru et al., 2004). Daily activity has specific pattern with two peaks: just after lights-on and around lights-off (Figure 1B). Morning peak is mostly driven by light, as in constant darkness (DD) it is much weaker, while evening peak is under circadian control. In addition, morning and evening anticipation is observed, which means that activity starts to increase around 3 h before the lights-on and lights-off (Figure 1B). Moreover, bimodal pattern of activity is observed also in clock mutants, but only in light-dark conditions, in constant darkness flies are completely arrhythmic. Clock mutants do not show morning anticipation, as they need light pulse to enhance activity level.

Compelling evidence attests to the influence of light on *Drosophila* rest-activity rhythms (recently reviewed in Helfrich-Förster, 2019). For instance, flies kept in constant darkness are sensitive to brief light pulses: they delay or advance their activity when the light stimulus is delivered in the early or late subjective night, respectively (Stanewsky et al., 1998). Also flies lacking compound eyes, *cli*^*eya*^ mutants (Helfrich-Förster et al., 2001), or with impaired photoreceptor signal transduction, due to deficiency in norpA-encoded phospholipase C-β activity (Bloomquist et al., 1988), have a clearly advanced evening activity (Schlichting et al., 2019a) and a similar phenotype has been recently reported in flies with degenerated photoreceptors (Cusumano et al., 2018; Niu et al., 2019; Weigelt et al., 2019).

Here we will review the role of light and light input pathways in shaping the fly sleep-wake pattern. In particular, we will initially describe neurotransmitters that regulate sleep in *Drosophila*. We will then focus on the sleep centers and pathways (visual and not visual) mediating light signal to the brain. Then, we will review the neuronal networks involving circadian pacemaker cells and finally the influence of light (timing and intensity) on sleep architecture.

## Sleep in *Drosophila*

As in mammals, sleep in insects is characterized by specific sleep posture and elevated sensory threshold. Although sleep patterns vary between different strains, sleep is always composed of daytime sleep, called “siesta,” with the maximum around noon, and nighttime sleep with peak at midnight (Figures 1C,D). Daytime sleep is less deep, with shorter single sleep episodes and lower arousal threshold (the level of sensory stimuli required for behavioural response), meaning that flies are more sensitive to awakening factors during the day than during the night (Hendricks et al., 2000; Huber et al., 2004). Wake/rest daily rhythms in *Drosophila* can be recorded by placing individuals in glass tubes and monitoring the movements using infrared beam-based activity monitors (DAMS, Trikinetics) or video recordings. Sleep in flies is defined as at least 5 min of total inactivity (Shaw et al., 2000), meaning that during this time no infrared break is recorded by the system. Recordings of local field potential in the brain suggest that *Drosophila* sleep can be divided to specific phases of different intensities, similar to mammalian sleep (Nitz et al., 2002; van Alphen et al., 2013; Raccuglia et al., 2019). Sleep differs according to sex: males sleep more, with comparable resting time during the day and night, while mated females sleep mostly during the night, and they are more active during the day (Huber et al., 2004). Sleep in *Drosophila* can be defined by the following parameters: bouts of sleep (number of sleep episodes), sleep bout length, which is useful for analysis of sleep fragmentation, and sleep latency/night offset (time between lights-off and the first sleep bout).

### Neurotransmitters

Sleep is controlled through neurotransmitters, divided into sleep-promoting [serotonin and gamma-aminobutyric acid (GABA)], wake-promoting (dopamine, octopamine, histamine) and those playing a dual role depending on target cells (acetylcholine, glutamate) (Table 1; reviewed in Ly et al., 2018). Both sleep-promoting neurotransmitters are released by dorsal pair medial neurons (DPMs) and directly affect mushroom bodies by inhibiting their activity (Haynes et al., 2015). GABA inhibits l-LNvs activity through Rdl receptor (Chung et al., 2009), and the pharmacological administration of GABA-A agonist (Gaboxadol) induces sleep behaviour in flies (Dissel et al., 2015) and humans (Faulhaber et al., 1997), indicating conserved role of GABA receptors in promoting sleep. Among wake-promoting molecules, dopamine and octopamine regulate the activity of sleep centers, central complex (CC), and mushroom bodies (MB) (Friggi-Grelin et al., 2003; Mao and Davis, 2009; Crocker et al., 2010), while histamine links retinal and extra-retinal photoreceptors to clock neurons (Oh et al., 2013). The role of octopamine is not well defined as recent data showed that the effect of octopamine could be sleep-promoting rather than wake-promoting (Deng et al., 2019). Finally, glutamate can promote sleep (Tomita et al., 2015) or wakefulness (Zimmerman et al., 2017) depending on the postsynaptic receptors. A similar effect is described for acetylcholine which promotes wakefulness by exciting l-LNvs when released from extra-retinal photoreceptors, Hofbauer–Buchner eyelets, and L2 neurons (McCarthy et al., 2011; Muraro and Ceriani, 2015; Schlichting et al., 2016), and has a sleep-promoting effect when released from mushroom bodies (Yi et al., 2013).

**TABLE 1:** Neurotransmitters involved in sleep regulation.

	Expression	Target cells	Receptor	References
Wake-promoting				
Dopamine	Sleep centers: MB PPL1, PPL3	Sleep centers: CC, FB l-LNvs MBON	D1-like (DopR1, DopR2) D2-like (DD2R)	Liu et al., 2012 Ueno et al., 2012 Sitaraman et al., 2015b Friggi-Grelin et al., 2003 Mao and Davis, 2009
Octopamine	APL	Visual system: Optic lobes Sleep centers: CC, MB l-LNvs protocerebrum, DILP2-expressed cells	Oamb Octß1R Octß2R Octß3R	Crocker et al., 2010
Histamine	Visual system: Photoreceptors, HB eyelets 18 cells in the brain	Visual system: Glial cells in the lamina, R1-8, LMC l-LNvs	HisCl1 Ort	Oh et al., 2013 Alejevski et al., 2019 Pantazis et al., 2008 Hamasaka and Nässel, 2006
Sleep-promoting				
Serotonin	DPM LBO5HT LMIo	Sleep centers: MB, FB LNvs Visual system: Epithelial glia, Mi, Mt, wide-field neurons in the lamina, wide field cells in the lobula and lobula plate	5-HT-1a 5-HT-2b 5-HT1b 5-HT-2a 5-HT-7	Yuan et al., 2005, 2006 Qian et al., 2017 Haynes et al., 2015
GABA	DPM, Visual system: C2, C3, amc, LMC (L1, L2), Mi4, CT1, TmY15	l-LNvs antennal lobes Sleep centers: MB, CC Visual system: large-field tangential neurons, C2, C3, LMC (L4)	GABAA (Rdl) GABAB (R1, R2, R3)	Chung et al., 2009 Haynes et al., 2015
Dual role				
Glutamate	Sleep centers: MB Visual system: LMC (L1), Tm (Tm9, Tm20), TmY, Mi (Mi9), Mt, Pm, Dm, Mi, bushy T (T), Tlp, Li, LPTC, LPi, amc	Visual system: large-field tangential cells, Mi9 Sleep centers: EB	iGluR (ionotropic), (Nmdar1, Nmdar2, GluRIIA-E, GluRIA, GluRIB) GluClα mGluR (metabotropic)	Tomita et al., 2015 Robinson et al., 2016 Raghu and Borst, 2011 Takemura et al., 2011 Liu and Wilson, 2013 Mauss et al., 2014, 2015 Sinakevitch-Pean et al., 2001
Acetylcholine	Sleep centers: MB Visual system: HB eyelets, LMC (L2, L4), amc, C2, Tm2	l-LNvs Visual system: LMC (L2, L4), Tm2	Muscarinic mAChR (A-C) Nicotinic nAChR (α1-7, β1-3)	McCarthy et al., 2011 Takemura et al., 2011 Buchner et al., 1986 Yasuyama and Salvaterra, 1999

*MB, mushroom bodies; FB, fan-shaped body; PPL, protocerebral posterior lateral cells; CC, central complex; MBON, mushroom body output neurons; APL, anterior paired lateral neurons; LMC, lamina monopolar cells; R, retina photoreceptors; DPM, dorsal pair medial; LBO5HT, large bilateral optic lobe 5-HTi neurons; LMIo, Lamina MIoinhibitory peptide-expressing cell; LNvs, ventral-lateral neurons; C2, C3, centrifugal cells; Tm, transmedullar cells; Amc, amacrine cells; TmY, transmedullar cells Y; Tlp, translobula plate cells; CT, complex tangential cell; Li, lobula intrinsic; cells; LPi, lobula plate intrinsic cells; Pm, proximal medulla cells; Dm, distal medulla cells; LPTC, lobula plate tangential cells; Mi, medulla intrinsic cells; Mt, medulla tangential cells.*

### Sleep Centers

**FIGURE 2:**
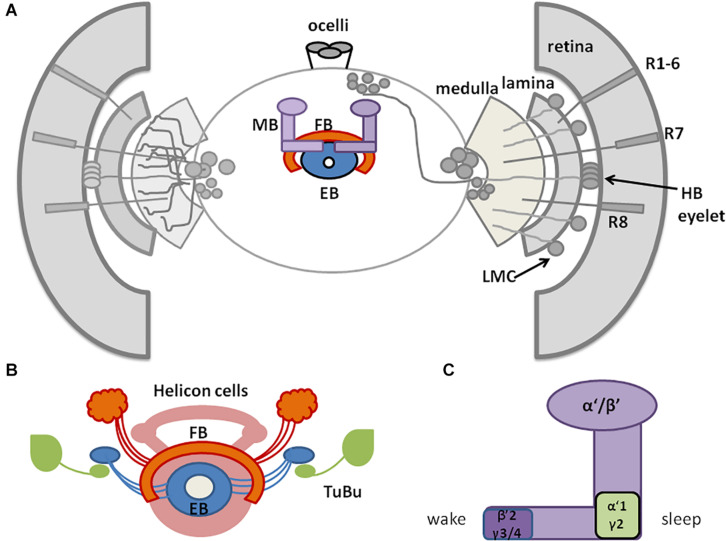
A schematic representation of *Drosophila* sleep centers and structures mediating light signals. **(A)** *Drosophila* brain with three photoreceptive structures: visual system (composed of retina and three optic neuropils: lamina, medulla, and lobula), extra-retinal Hofbauer–Buchner (HB) eyelets and ocelli, located on the top of head. Light is received by retinal photoreceptors R1–6, which terminate in the lamina, and R7–8, terminating in the medulla and transmitting through lamina monopolar cells (LMC) to the deep brain. HB eyelets, located between retina and lamina, are sensitive to high intensity light and transmit signal directly to clock neurons (ventral-lateral neurons_LNvs). In the central brain sleep centers are located with mushroom bodies (MB), ellipsoid body (EB), and fan-shaped body (FB). **(B)** Scheme of sleep center, composed of fan-shaped body (FB), mushroom bodies (MB), ellipsoid body (EB), and additional cells: tubercular bulbar neurons (TuBu) and helicon cells. **(C)** Mushroom bodies are composed of vertical (α, α’) and horizontal (β, β’, γ) lobes. Wake-promoting neurons are located in β’2, γ3/4, while sleep-promoting ones in α’1, γ2 region of MB.

*Drosophila* sleep centers are located in different brain regions, although the most essential ones reside in the central and dorsal region, as MB and CC, composed of dorsal fan-shaped body (FB), and ellipsoid body (EB) with the ring structure (EB-R2) (Joiner et al., 2006; Pitman et al., 2006; Donlea et al., 2011; Liu et al., 2012, 2016; Guo et al., 2016; Figures 2A,B).

Mushroom bodies are composed of neurons called Kenyon cells (Technau, 2007), whose axons form lobes: two vertical (α, α’) and three horizontal (β, β’, γ). MB contains both wake-promoting and sleep-promoting neurons, located in β’2, γ3/4 and α’1, γ2 region, respectively (Joiner et al., 2006; Sitaraman et al., 2015b; Artiushin and Sehgal, 2017; Figure 2C). MB receive inhibiting, wake-promoting signals through serotonin and GABA (Yuan et al., 2006; Haynes et al., 2015). Specific MB compartments send information to mushroom body output neurons (MBONs) *via* glutamate and acetylcholine, that have a wake- or sleep- promoting effect, respectively (Aso et al., 2014; Sitaraman et al., 2015a). MB express additional sleep-promoting factors. Among these, Neurocalcin (NCA) and Noktochor (Nkt) promote nighttime sleep by suppressing nocturnal arousal (Chen et al., 2019; Sengupta et al., 2019).

**FIGURE 3:**
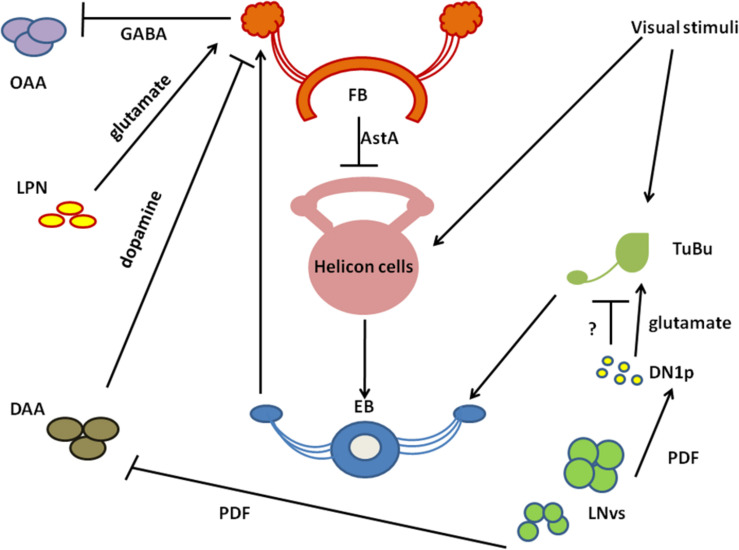
A schematized circuit of neurotransmission regulating sleep centers. Light affects clock neurons (ventral-lateral neurons_LNvs) activity, which stimulates pigment dispersing factor (PDF) release. In turn, PDF inhibits dopamine arousal neurons (DAAs), which normally decrease fan-shaped body (FB) activity through dopamine. Lack of dopamine signalling promotes sleep. PDF activates also Dorsal Neurons posterior (DN1sp), which can inhibit tubercular bulbar cells (TuBu), or activate them through glutamate. Visual stimuli affect Tubu and helicon cells activity, both send signals to the ellipsoid body (EB). EB coordinates FB activity and receives feedback signals from FB through helicon cells, which are inhibited by AstA released from FB. In addition, FB obtains signals from lateral posterior neurons (LPNs) through glutamate. FB processes all inputs and sends final sleep-promoting information through inhibiting GABA signalling to output arousal neurons (OAAs).

The CC is involved in the regulation of locomotor activity and visual processing (Liu et al., 2006; Poeck et al., 2008; Triphan et al., 2010; Seelig and Jayaraman, 2013). The upper part of CC contains the FB, with sleep-promoting ExFl2-cells, which receive signals from the protocerebral posterolateral cluster 1 (PPL1) and protocerebral posteromedial 3 (PPM3) (Liu et al., 2016). This dopaminergic pathway inhibits FB activity and suppresses sleep (Liu et al., 2012; Ueno et al., 2012; Kayser et al., 2014; Pimentel et al., 2016; Ni et al., 2019). FB can be also activated by glutamatergic input from the circadian clock, represented by Allatostatin A (AstA)-expressing LPN cells (Ni et al., 2019). Both inputs are integrated in FB to precisely control its activity and ultimately regulate shifts between sleep and wake states. In the final step, active FB releases GABA, which inhibits octopaminergic output arousal neurons (OAA), thus promoting sleep (Ni et al., 2019; Figure 3).

Below the FB, EB is located, which is involved in memory formation and startle response to mechanical stimulation. The major neurons composing EB are called ring neurons (R), and they receive synaptic signals from the anterior visual tract, through tubercular bulbar (TuBu) neurons (Omoto et al., 2017). Additional cells, called helicon cells, receive and integrate visual inputs and connect FB and EB: during wakefulness, helicon cells are sensitive to visual inputs and propagate signals to R2 cells, which are important for the regulation of sleep depth. R2 cells activate sleep-promoting ExFl2 neurons, thus increasing sleep need. In turn, during sleep AstA released by FB inhibits helicon cells, which causes decreased responsiveness to visual stimuli (Donlea et al., 2018; Figure 3).

## How Do Light Inputs Modulate Sleep in *Drosophila*?

Light signals to *Drosophila* brain are mediated either by visual structures, such as two large compound eyes (retinal photoreceptors) and two Hofbauer–Buchner eyelets (HB eyelets, extraretinal photoreceptors), and non-visual pathways, involving three ocelli and deep brain photoreceptors CRYPTOCHROME (CRY), QUASIMODO (QSM), and Rhodopsin 7 (Rh7) (reviewed in Helfrich-Förster, 2019).

### Compound Eyes

*Drosophila* compound eyes comprise ∼800 units, called ommatidia, organized in regular structures innervating and conveying visual signals to the four distinct neuropil regions of the optic lobe (lamina, medulla, lobula, and lobula plate). Each ommatidium houses 20 cells, eight of which are photoreceptors (R1–R8) designated in processing light inputs as function of position, spectral composition, and axonal projections. The six outer photoreceptors (R1–R6), expressing the broad spectrum rhodopsin (Rh1) (Hardie, 1979; O’Tousa et al., 1985; Zuker et al., 1985), project to the lamina (Braitenberg, 1967; Strausfeld, 1971) and are intended for motion detection and image formation (Heisenberg and Buchner, 1977; Yamaguchi et al., 2008). The two inner central photoreceptors (R7–R8) reach and innervate the distant medulla and participate in color, UV, and polarized light detection (Melamed and Trujillo-Cenóz, 1967; Montell, 2012). R7–R8 photoreceptors are clustered in 30% “pale” [R7 expressing UV-sensitive-Rh3 (331 nm) and R8 expressing blue-sensitive-Rh5 (442 nm)] and 70% “yellow” ommatidia [R7 expressing UV-sensitive-Rh4 (355 nm) and R8 expressing green-sensitive-Rh6 (515 nm)] (Fryxell and Meyerowitz, 1987; Montell et al., 1987; Zuker et al., 1987; Feiler et al., 1992; Chou et al., 1996, 1999; Huber et al., 1997; Papatsenko et al., 1997; Salcedo et al., 1999). A seventh Rhodopsin (Rh7) (Adams et al., 2000) is expressed also in the compound eyes (specifically in R8) as well as in other brain neurons (including some clock neurons) (Senthilan and Helfrich-Förster, 2016; Grebler et al., 2017; Kistenpfennig et al., 2017; Ni et al., 2017; Senthilan et al., 2019). Its role and expression pattern have been recently discussed (Senthilan et al., 2019).

The visual cascade complex is located in the rhabdomeres of photoreceptor cells: the G-protein (Gq) activates the phospholipase C (PLC), [encoded by *norpA* (Bloomquist et al., 1988; Scott et al., 1995)] which hydrolyzes phosphatidylinositol 4,5-bisphosphate (PIP_2_) and promotes the opening of the TRP and the TRP-like (TRPL) cation channels (reviewed in Montell, 2012). The triggered calcium current is then balanced by the Na^2 +^ /Ca^2+^ exchanger, Calx (Wang et al., 2005).

Each outer photoreceptor cell forms a tetrad synapse with L1 and L2 laminar neurons and L3 or amacrine cell or epithelial glia. Under certain conditions (bright light and impaired synaptic transmission) lamina interneurons feed back to photoreceptor cells, modulating their output (Zheng et al., 2006). In the lamina, projections from R1–6 are organized in synaptic modules called cartridges, in which three epithelial glial cells surround six photoreceptor terminals with invaginating capitate projections (Stuart et al., 2007). On the other hand, inner photoreceptors and lamina neurons form synaptic modules (columns) with medulla interneurons and neurons that convey visual information to the lobula and lobula plate neuropils (Stark and Carlson, 1986; Meinertzhagen and O’Neil, 1991; Perez and Steller, 1996; Rivera-Alba et al., 2011; Millard and Pecot, 2018). Recently, a new class of *Drosophila* interneurons has been discovered: the Allatostatin C (AstC)/crustacean cardioactive peptide receptor (CcapR) expressing neurons, which convey light input from the compound eyes directly to the circadian pacemaker neurons, through the accessory medulla (aMe) (Li et al., 2018). The medulla thus receives and processes both motion and colour information coming from different retinotopic maps (Rister et al., 2007; Gao et al., 2008).

**FIGURE 4:**
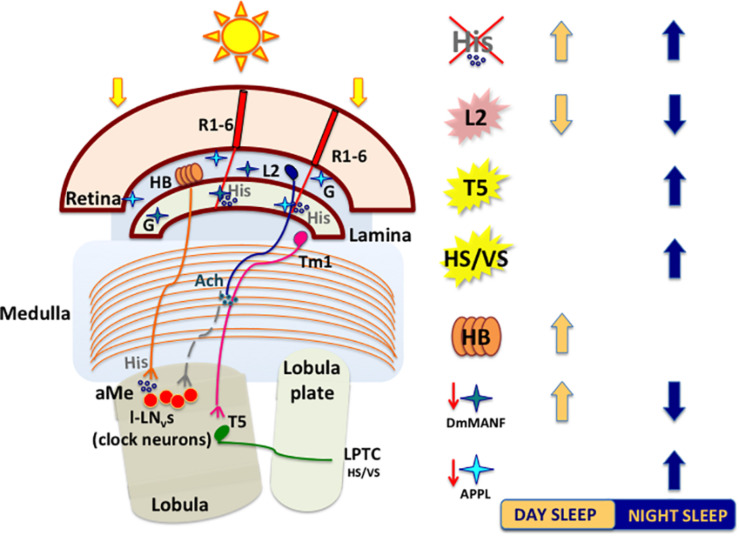
A schematized and simplified circuit showing how light perceived by retinal and extra-retinal structures impacts sleep. Light-activated retinal photoreceptors release histamine (His). Mutations affecting the histamine biosynthesis pathway (*hdc* mutants) lead to increase in sleep. R1–R6 photoreceptors convey signals to cholinergic large monopolar laminar cells (L2) that connect indirectly both clock neurons (l-LNvs) and neurons involved in motion pathways (T5). Temperature-Sensing TRP Channel activation in L2 neurons strongly suppresses sleep by releasing ACh that, in turn, promotes the bursting of l-LNvs. T5 cells axons transfer motion information to various types of lobula plate tangential neurons (LPTCs), like HS (horizontal motion) and VS (vertical motion) neurons, to further reach the central brain. Optogenetic activation of motion processing neurons (T5 or HS/VS) increases nighttime sleep. The extraretinal photoreceptors (HB eyelets) release histamine in the accessory Medulla (aMe), preventing l-LNvs from firing and thus increasing siesta during the day. Glia cells in lamina interact with photoreceptors and shape the wake/sleep pattern of flies. Downregulation of *DmMANF* (mesencephalic astrocytes-derived neurotrophic factor) in epithelial glia induces lamina neurodegeneration and leads to increase in daytime sleep. Downregulation of *Appl* (amyloid precursor protein like) in glia cells increases nighttime sleep. Light blue (APPL) and dark blue (DmMANF) stars indicate cortex and epithelial lamina glia, respectively. L2: heat-activated (pink); T5 and HS/VS: optogenetically activated (yellow). G, glia cell; His, histamine; Ach, acetylcholine.

The epithelial glial cells surrounding lamina cartridges express the Mesencephalic Astrocyte-derived Neurotrophic Factor DmMANF, orthologue of mammalian MANF and CDNF (cerebral dopamine neurotrophic factor), involved in supporting the survival of dopaminergic neurons (Lindholm and Saarma, 2010). DmMANF is also involved in the maintenance of dopaminergic neurons, as *DmMANF* null mutants display extremely low levels of dopamine and decreased dopaminergic neurites (Palgi et al., 2009). In the adult fly, DmMANF is also expressed in the retina, specifically in the photoreceptor cell bodies, and in the lamina (lamina cortex and synaptic neuropil) (Stratoulias and Heino, 2015). At structural level, the silencing of *DmMANF* in glial cells induces degeneration of the lamina, in particular in the lamina epithelial glial cells, which exhibits holes and/or tightly packed membranes and also a decrease of capitate projections in the cartridges (Walkowicz et al., 2017). In glial cells, DmMANF is also involved in controlling the levels of dopamine and other neurotransmitters responsible for *Drosophila* behaviour. In fact, downregulation of *DmMANF* in glia alters the sleep/activity pattern of flies in LD, with decreased activity in the light phase and increased activity in the dark phase of the cycle (Walkowicz et al., 2017). Conversely, these flies display a reduction of nightime sleep and a slight increase of sleep in the early day (Figure 4). The sleep modulating role of DmMANF is supported by the significant upregulation of transcripts involved in the dopamine synthesis pathway observed in hypomorphic *DmMANF* mutant embryos (Palgi et al., 2012).

Glial cells express the Amyloid Precursor Protein-Like (APPL), known for its crucial role in neuronal physiology and cellular biology and its involvement in age-dependent behavioural deficits and neurodegeneration, as consequence of production and deposition of toxic β-amyloid peptides, in both mammals and flies (Carmine-Simmen et al., 2009). In *Drosophila*, this is true also for the glial cells in the subretinal layer of lamina cortex, where the correct cleavage of APPL is fundamental for their survival. Indeed, loss of function mutation or knock-down of the beta-site Amyloid Precursor Cleaving Enzyme (dBACE) in photoreceptor neurons result in glial cell death and progressive lamina degeneration (Bolkan et al., 2012).

The role of APPL in glia is not only related to its neurotoxic effects, as it has been recently shown to be involved in the physiology and regulation of sleep/wake cycles (Farca Luna et al., 2017). The downregulation of *Appl* in cortex and astrocyte-like glia significantly increases nighttime sleep, which exhibits longer sleep-bouts (Figure 4) and, conversely, the overexpression of *Appl* in these cells results in reduced sleep amount and increased sleep latency (Farca Luna et al., 2017). This effect on sleep/wake regulation is due to an altered glutamate recycling, as the downregulation of *Appl* increases the expression of genes involved in reuptake and recycling of the neurotransmitter, such as the glutamate transporter *excitatory amino acid transporter 1* (*dEaat1*) and the *glutamine synthetase* (*Gs*) (Farca Luna et al., 2017). Moreover, the downregulation of *Appl* changes also the cellular distribution of Innexin 2 (Inx2), highly expressed in the layers of laminar pseudocartridge and satellite glia, where it plays a fundamental role in modulating the level of carcinine, and therefore histamine, essential for a proper visual synaptic transmission (Chaturvedi et al., 2014).

As previously mentioned, R1–6 photoreceptors are also involved in visual motion, that is the detection of direction-selective signals, fundamental for fly survival. The luminance information from R1–6 is integrated by some large motion-sensitive neurons in the lobula plate, called lobula plate tangential cells (LPTCs), specific for vertical or horizontal motion (VS and HS, respectively) and responding by selective hyperpolarization or depolarization (reviewed in Borst et al., 2020). Each lamina cartridge specifically conveys brightness increments or decrements information to subsets of downstream motion detecting neurons, *via* a specific set of cells in the medulla, called trans-medulla Y (TmY). In particular, L1 pathway conveys luminance increments to specific layers of the lobula (T4 cells-ON channels), while the L2–4 pathway transmits information about brightness decrements to the lobula plate (T5 cells-OFF channels) (Borst et al., 2020). Axon terminals from T4 and T5 neurons then connect to the dendrites of LPTCs (HS and VS) in the lobula plate (Borst et al., 2020), from where the information is further transmitted to the central brain likely through descending neurons (Suver et al., 2016). LPTCs also receive direction information from another source. Indeed, T4 and T5 cells contact and send an inhibitory glutamatergic signal to a group of neurons in the lobula plate, the bi-stratified lobula plate intrinsic (LPi) cells, that convey this signal to the tangential cells expressing glutamatergic Cl^–^ channel α (reviewed in Borst et al., 2020).

Visual information processed by motion circuits play an important role in sleep regulation. Flies lacking HS and VS neurons (*omb*^*H*31^ mutants) display a reduced and fragmented sleep compared to wild-type (wt), while the optogenetic activation of these cells results in an increase of nighttime sleep (Kirszenblat et al., 2019). Moreover, the optogenetic activation of T5 neurons leads to a consolidation of nighttime sleep, with increased bout duration and lower bouts number (Kirszenblat et al., 2019; Figure 4).

#### Histamine, the Major Neurotransmitter in the Compound Eyes

Histamine is the most important neurotransmitter released by the compound eyes (Hardie, 1987, 1989), and histamine-immunoreactivity has been detected in the optic lobes, in neurons adjacent to LNs and DNs, ocelli, in the eyelets axons, in 18 cell bodies in protocerebrum (HP1–4) and 2 cell bodies in the subesophageal ganglion (Nässel, 1999; Hamasaka and Nässel, 2006; Hong et al., 2006; Oh et al., 2013). The biogenic amine is synthetized in photoreceptors, from L-histidine, by the histidine decarboxylase (Hdc) and flies deficient for this enzyme activity (*hdc*^*P*218^) have disrupted photoreceptor synaptic transmission (Burg et al., 1993). Light-depolarization of retinal photoreceptors triggers the fast release of histamine to the downstream lamina monopolar neurons; this, in turn, opens the histamine-gated chloride channels and leads to hyperpolarization (Wang and Montell, 2007; Pantazis et al., 2008). Electroretinograms in postsynaptic lamina neurons record ON and OFF transient peaks as a function of light (Alawi and Pak, 1971; Heisenberg, 1971). The epithelial glia cells surrounding synaptic cartridges work in coordinating photoreceptor-glia communication in the lamina: in fact, in capitate projections histamine is conjugated to β-alanine by Ebony, to form β-alanylhistamine (carcinine) (Stark and Carlson, 1986; Meinertzhagen and O’Neil, 1991; Borycz et al., 2002; Richardt et al., 2002, 2003; Hartwig et al., 2014). Carcinine is then transported back to photoreceptors by the transporter CarT (Stenesen et al., 2015; Xu et al., 2015; Chaturvedi et al., 2016) and cleaved again into histamine and β-alanine by Tan (Borycz et al., 2002; Wagner et al., 2007). Interruption of this cycle results in the loss of visual transmission (Rahman et al., 2012).

In *Drosophila*, histamine gates two chloride channels: the outer rhabdomeres transientless (ort) and histamine-gated chloride channel subunit 1 (HisCl1) (Gengs et al., 2002; Gisselmann et al., 2002; Witte et al., 2002; Zheng et al., 2002). Ort is expressed in lamina (L1–L3 cells), medulla, lobula neuropils, ocellar postsynaptic interneurons, Pars Intercerebralis (PI), FB, cells in the lateral and central brain and thoracic ganglia (Hong et al., 2006; Gao et al., 2008; Pantazis et al., 2008; Lin et al., 2016; Schnaitmann et al., 2018). In lamina interneurons, it plays a key role in transmitting motion detection inputs coming from retina photoreceptors: its overexpression in L1 and L2 can restore the ON and OFF transients in electroretinograms and motion detection responses lost in *ort*-null mutants (Gengs et al., 2002; Rister et al., 2007; Gao et al., 2008; Pantazis et al., 2008). HisCl1 receptor is strongly expressed in lamina epithelial glial cells surrounding cartridges, in neurons in the medulla (Gao et al., 2008; Pantazis et al., 2008), in R7 and R8 photoreceptors (Tan et al., 2015; Schnaitmann et al., 2018; Alejevski et al., 2019; Davis et al., 2020) and many other cell types, including the large LNvs (Hamasaka and Nässel, 2006; Hong et al., 2006).

Histamine released by light-activated photoreceptors likely acts in at least two different pathways directly involved in sleep regulation: the visual (photic) input and the motion detection pathways, distinct signalling dynamics both relying on activation of lamina interneurons L2 (Meinertzhagen and O’Neil, 1991; Meinertzhagen and Sorra, 2001; Shinomiya et al., 2014, 2019; Muraro and Ceriani, 2015; Kirszenblat et al., 2019; Borst et al., 2020; Figure 4).

In mammals, histamine is known to play a wake-promoting role (Thakkar, 2011), that seems to be conserved in insect. In *Drosophila*, histamine treatment causes sleep time reduction (Oh et al., 2013), while administration of its receptor antagonist increases sleep (Shaw et al., 2000). Moreover, mutations in the *hdc* gene [*hdc*^*P*211^ and *hdc*^*P*218^ (Burg et al., 1993)], lead to a significant increase of daytime sleep duration and number of sleep episodes in comparison to wt (Oh et al., 2013; Figure 4). Similar data obtained in constant darkness indicate that the observed wake-promoting effect depends on histamine, and it is not connected with defects in photoreception in the eye (Oh et al., 2013). Of the two histamine receptors, only HisCl1 located on the surface of l-LN_*v*_s is involved in sleep regulation (Oh et al., 2013).

### Ocelli

Ocelli complex is composed of three ocellar cells, interocellar cuticle and bristles (Haynie and Bryant, 1986). They contain 80–100 photoreceptors expressing the UV-sensitive Rhodopsin2 (Mismer and Rubin, 1987; Feiler et al., 1988; Pollock and Benzer, 1988). The role of ocelli is to adjust sensitivity of the compound eyes (Hu and Stark, 1980) and to collect information about the horizontal position (Krapp, 2009). They also contribute to entrainment to long and short days (Rieger et al., 2003), *via* a norpA-independent pathway (Saint-Charles et al., 2016). They use histamine as neurotransmitter, and they do not contact directly with clock neuron processes (Hamasaka and Nässel, 2006). A specific role for this structure in sleep has not been reported yet.

### Hofbauer–Buchner Eyelets: Direct Light Signalling to the Pacemaker

Hofbauer–Buchner eyelets originate from the larval visual system, called Bolwig organs (BO), involved in the regulation of many light-dependent behaviours (Busto et al., 1999; Hassan et al., 2000). Larval BO is cholinergic (Yasuyama and Salvaterra, 1999), but it uses norpA-dependent phototransduction pathway, similar to retinal photoreceptors (Busto et al., 1999; Hassan et al., 2000). It is composed of 12 cells: eight of them express Rh6 and 4 of them express Rh5 (Sprecher and Desplan, 2008), and their projections terminate in the area of LNvs (Kaneko et al., 1997). Rh6-expressing cells die during development, and four others switch expression from Rh5 to Rh6 (Sprecher and Desplan, 2008). Adult HB express Rh6 and are sensitive to 480 nm wavelength (Helfrich-Förster et al., 2002), yet there are evidences that they may use an alternative mechanism of phototransduction, norpA-independent, like cascade described by Chang and Ready (2000). Although Rh5 expression in HB could not be detected by immunostaining (Yasuyama and Meinertzhagen, 1999; Malpel et al., 2002), the expression of GFP under the Rh5-Gal4 driver was revealed as a weak signal (Malpel et al., 2002). In the adult, HB act as circadian photoreceptive organs (Hofbauer and Buchner, 1989; Yasuyama and Meinertzhagen, 1999) and contribute to the synchronisation of circadian clock, in terms of entrainment to long and short days (Helfrich-Förster et al., 2001, 2002; Rieger et al., 2003). At the molecular level, they are involved in synchronisation of TIM and PER expression in s-LNvs (Helfrich-Förster et al., 2001), l-LNvs and DN1s (Mealey-Ferrara et al., 2003; Veleri et al., 2007).

**FIGURE 5:**
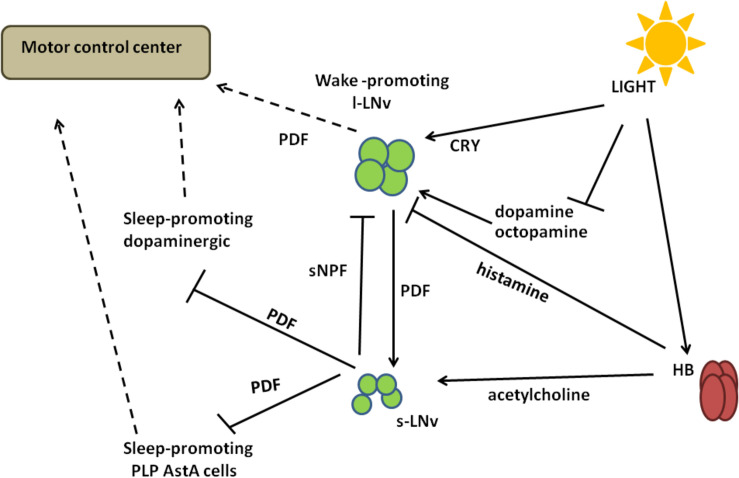
A schematized circuit of circadian regulation of sleep. Light signals received by Hofbauer–Buchner eyelets (HB) are transmitted through acetylcholine to small ventral-lateral neurons (s-LNvs) and through histamine to large ventral-lateral neurons (l-LNvs). Activated s-LNvs release pigment dispersing factor (PDF), which inhibits dopaminergic and AstA-expressing sleep-promoting cells. The regulation of l-LNvs activity is more complex, as they express light-sensitive Cryptochrome (CRY) as well as dopamine and octopamine receptors. Light activates CRY, but at the same time it inhibits the response to dopamine and octopamine, decreasing signal inputs to l-LNvs. In addition, l-LNvs receive inhibiting inputs from s-LNvs through short Neuropeptide F (sNPF). Different signalling inputs are processed in l-LNvs and transmitted to the motor control center through PDF.

Hofbauer–Buchner axons terminate in the accessory medulla and they can directly contact with pigment dispersing factor (PDF)-expressing LNvs in aMe (Helfrich-Förster et al., 2002; Malpel et al., 2002). Eyelets express both histamine and acetylcholine as neurotransmitters (Hofbauer and Buchner, 1989; Pollack and Hofbauer, 1991; Yasuyama and Meinertzhagen, 1999; Damulewicz et al., 2020; Figures 4, 5).

Light signals received by HB eyelets in the morning are transmitted *via* acetylcholine and excite s-LNvs *via* nicotinic receptors (Wegener et al., 2004; McCarthy et al., 2011; Schlichting et al., 2016), causing increased cAMP levels (Lelito and Shafer, 2012) and a wake-promoting effect. At the same time, histamine released from HB inhibits l-LNvs (Schlichting et al., 2016). In the morning l-LNvs are less active, with decreased firing observed. Then they start to accumulate Ca^2+^ that reaches its maximal level around midday (Liang et al., 2016), when they could receive input from other cells, that is, from L2 cells in the medulla (Muraro and Ceriani, 2015). l-LNvs increase firing and release PDF to activate evening cells, ultimately increasing the evening activity.

### Deep Brain Photopigments

Cryptochrome (CRY) is a blue-light-sensitive protein (VanVickle-Chavez and Van Gelder, 2007) playing many different roles, ranging from photoreceptor to magnetoreception and metabolism regulation (for review see Damulewicz and Mazzotta, 2020). It is expressed in a broad range of cells in the brain: in circadian pacemaker neurons (all five s-LNvs, l-LNvs, three of the six LNds, and some of the DN1s), but also in non-clock neurons, glia and visual system (Benito et al., 2008; Yoshii et al., 2008; Damulewicz and Pyza, 2011; Fogle et al., 2011). Its photoreceptive role allows the entrainment of molecular clock to environmental light conditions through conformational changes that expose specific domains and promote binding TIM or PER (Ceriani et al., 1999; Koh et al., 2006; Peschel et al., 2009; Rosato et al., 2001), targeting TIM to ubiquitination and degradation in proteasomes (Peschel et al., 2009). The role of CRY in the visual system is more complex, as it plays a role of circadian transcriptional repressor (Collins et al., 2006), in maintaining the proper localization of phototransduction cascade complex (Mazzotta et al., 2013; Schlichting et al., 2018) and in enhancing photosensitivity during the night (Damulewicz et al., 2017; Mazzotta et al., 2018).

Quasimodo (QSM) is a light-sensitive protein, belonging to the extracellular membrane-anchored Zona pellucida (ZP) domain family. It is expressed in all clock neuronal groups, except for LPNs, however not in every cell within the cluster. Most of the clock cells co-express both, QSM and CRY, but some of them, like DN2s and DN3s, do not express CRY, suggesting that QSM works in a CRY-independent pathway. In addition, QSM is expressed in non-clock cells, located in close proximity to the pacemaker (Chen et al., 2011). Light exposure increases QSM levels inside the cell, *via* a post-translational mechanism that involves the extracellular ZP domain light-dependent cleavage (Plaza et al., 2010; Buhl et al., 2016). Active QSM was proposed to change membrane conductance following interaction with the Na^+^, K^+^, Cl^–^ co-transporter (NKCC) and the Shaw K^+^ channel (dKV3.1) (Buhl et al., 2016). QSM regulates electrical excitability also in clock neurons: it modulates l-LNvs daily changes of activity, as its downregulation results in a constitutively more active state, similar to that observed during the day time, while *qsm* overexpression leads to a constitutive less active, night-like state (Buhl et al., 2016). Moreover, QSM is involved in the light-dependent TIM degradation process and it can affect TIM stability in a CRY-independent pathway (Chen et al., 2011).

### Circadian Pacemaker Neurons

Sleep timing and duration are highly influenced by the circadian clock, which promotes the consolidation of sleep during the night in diurnal species, such as *Drosophila* and human, and during the day in nocturnal animals, such as rodents (Kunst et al., 2014; Liu et al., 2014). Indeed, in flies lacking the main clock genes *period* and *timeless*, the sleep episodes are randomly distributed across the 24 h, although the mean rest levels do not differ from wt (Hendricks et al., 2000). Moreover, flies mutants for both *Clock* and *cycle* show a significant decrease in daily consolidated rest in DD conditions, with brief rest and prolonged activity bouts (Shaw et al., 2002; Hendricks, 2003), and *cyc*^01^ mutants show also an excessive response to sleep deprivation, with a persistent large increase in sleep (Shaw et al., 2002).

#### PDF Expressing LNvs

##### LNvs and serotoninergic signalling: modulation of the circadian light sensitivity

The serotoninergic pathway regulates many aspects of behaviour, including sleep/wake cycles (Ursin, 2002). In *Drosophila*, it positively controls sleep and negatively modulates circadian photosensitivity: treatment with the serotonin precursor 5-hydroxyl-L-tryptophan (5-HTP) results in significant increase of sleep amount and reduction of the light-induced phase shift, especially in response to high intensity light pulses (Yuan et al., 2005, 2006). This dual role is mediated by two distinct receptors, d5-HT1A and d5-HT1B, sharing high homology with the mammalian counterpart (5-HT1A), that controls many aspects of animal behaviour, including sleep (Boutrel et al., 2002; Yuan et al., 2005).

*d5-HT1A* is highly expressed in the MB, at levels that remain constant during the day (Yuan et al., 2006). d5-HT1A is specifically involved in regulating sleep amount and consolidation, as flies carrying a deleted form of d5-HT1A exhibit a significant reduction and fragmentation in sleep, with nighttime sleep bouts reduced in length but increased in number (Yuan et al., 2006). This behaviour is specifically dependent on d5-HT1A in MB, since it can be completely rescued by overexpression of the receptor in these neurons (Yuan et al., 2006). Treatment with 5-HTP increases sleep in *d5-HT1A* mutant flies, indicating that other unidentified serotonin receptors are also involved in sleep regulation (Yuan et al., 2006).

d5-HT1B is expressed in different brain structures, including LNvs (Yuan et al., 2005). The expression of d5-HT1B in the adult fly brain does not show circadian oscillation, neither as mRNA nor as protein, but its levels are influenced by the clock, as they appear to be upregulated in *tim*^01^ and downregulated in *cyc*^0^ mutants (Yuan et al., 2005).

In clock cells, d5-HT1B is involved in modulating the circadian light sensitivity: flies overexpressing this receptor in the clock neurons exhibit a reduced magnitude of the response to phase shift following light pulse, mirrored by a reduced light-dependent TIM degradation (more evident in s-LNvs compared to l-LNvs). Conversely, the downregulation of d5-HT1B results in an increased phase shift, also toward low light intensities (Yuan et al., 2005).

Serotonin and d5-HT1B effects on circadian light sensitivity are related to the CRY signalling pathway: while the overexpression of d5-HT1B in a wt background induces increased levels of rhythmicity in constant light, the overexpression of d5-HT1B in a *cry* mutant background (*cry*^*b*^) has no effect on the rhythmicity exhibited by *cry*^*b*^ flies (Yuan et al., 2005).

##### LNvs and Neuronal Structural Remodeling

Remodeling of neuronal connections is fundamental for the neuronal circuits to detect environmental changes and drive complex behaviour. In *Drosophila*, the circadian behaviour also results from a clock-controlled structural plasticity that contributes to the transmission of information downstream of pacemaker neurons (Fernández et al., 2008).

PDF positive LNvs rhythmically express the miRNA *miR-210* (Chen and Rosbash, 2017), that plays an important role in the phasing of the circadian locomotor activity (Cusumano et al., 2018; Niu et al., 2019). *miR-210* is also involved in the regulation of sleep levels and temporal distribution, and this role is likely correlated to the morphology remodeling of l-LNvs: in fact the *miR-210* overexpression in clock cells results in a significant increase in daytime sleep and a dramatic alteration of l-LNvs morphology and projections (Cusumano et al., 2018).

Small ventral-lateral neurons express dTau, a protein with microtubule-binding properties, homolog to mammalian Tau, known to be involved in the maturation and establishment of synaptic networks regulating complex behaviours (Abruzzi et al., 2017; Tracy and Gan, 2018). dTau plays an important role in shaping behavioural rhythms and sleep patterns: in either LD cycles or DD, *dTau* mutant flies exhibit an increased activity during the day/subjective day, more pronounced in the middle of the day, when wt flies have a “siesta” (Arnes et al., 2019). This altered locomotor phenotype is mirrored by pronounced sleep alterations: *dTau* null flies exhibit a significant alteration of daytime sleep, while nocturnal sleep is not affected: the total daytime sleep is significantly decreased, including the “siesta,” the sleep episodes are shorter, that is, sleep is more fragmented, and the sleep latency is significantly longer (Arnes et al., 2019).

At the neuronal level, dTau plays an essential role in modulating the structural plasticity of s-LNvs terminals: in wt flies the dorsal projections of s-LNvs neurons display a rhythmic remodeling, with significantly higher degree of axonal arborisation in the early day (ZT2) compared to early night (ZT14) (Fernández et al., 2008). *dTau* null flies exhibit a significant reduction in the structural morphology of the s-LNv at ZT2 compared to wt, in line with the behavioural defects (increased activity and decreased sleep) displayed in the early day (Arnes et al., 2019). Furthermore, in s-LNvs, dTau shows rhythmic expression at both mRNA and protein levels, with significantly higher levels in the early morning (ZT2) than in the early night (Abruzzi et al., 2017; Arnes et al., 2019). This temporal rhythmic pattern perfectly matches with its role in modulating the structural plasticity of s-LNvs terminals (Arnes et al., 2019).

#### Large Ventral-Lateral Neurons: The Heart of the Sleep Circuit

Large ventral-lateral neurons are among the first clock neurons that have been identified (Zerr et al., 1990) and they have a predominant role in detecting light and transferring the photic information to the circadian clock (reviewed in Helfrich-Förster, 2019). By using different signalling pathways l-LNvs integrate light stimuli and produce appropriate behavioural responses (Figure 5).

##### l-LNvs are directly activated by light

Large ventral-lateral neurons display an acute increase in their firing rate in response to light, and this altered electrical activity influences locomotor behaviour, sleep and arousal (Sheeba et al., 2008a). l-LNvs hyperexcited flies exhibit an increase in nocturnal activity compared to controls, mirrored by a disruption in the quantity and quality of nocturnal sleep (Sheeba et al., 2008a). Moreover, the increased nocturnal behaviour of l-LNvs hyperexcited flies is mediated by a PDF-dependent mechanism, as *Pdf* mutants exhibit a nocturnal activity significantly lower compared to wt (Sheeba et al., 2008a). The light-induced firing rate of l-LNvs is dependent on the presence of the circadian photoreceptor CRY, highly expressed in these clock cells (Emery et al., 2000). Indeed, in *cry*^*b*^ hypomorphic mutants, the electrophysiological response is attenuated (Sheeba et al., 2008b), while it is completely abolished in *cry*-null flies (Fogle et al., 2011). Conversely, the light-induced firing of l-LNvs is functionally rescued by targeted expression of CRY in the l-LNvs.

Large ventral-lateral neurons are part of the peptidergic arousal pathways in *Drosophila*. The hyperactivation of these cells by overexpression of NaChBacGFP, a bacterial-derived voltage-gated sodium channel (Nitabach et al., 2005), results in a dramatic increase of nighttime activity and, by a genetic manipulation, it has been also shown that the stimulation of l-LNvs is sufficient to promote arousal at night (Shang et al., 2008). Moreover, l-LNvs-mediated arousal is light-dependent: flies in which this subset of clock cells is genetically ablated exhibit an increased sleep in LD, even more evident in LL, a phenotype completely lost when flies are moved to DD (Shang et al., 2008). Another important feature of l-LNvs is that they signal light information to the circadian clock at dawn: indeed, l-LNvs-deficient flies exhibit no phase advance response to light at ZT21 compared to control, while no differences between the two genotypes are observed for light pulse at ZT15 (Shang et al., 2008).

Pigment dispersing factor is specifically involved in increasing flies’ activity in the late night: *Pdf*^01^ mutants, as well as flies with null mutation in the receptor for PDF (*Pdfr*^*han*5304^), exhibit an increased sleep during the late night, while flies in which the PDF-expressing neurons are genetically ablated, show a prolonged sleep (Chung et al., 2009). The lack of PDF-mediated signalling is partially compensated by light: in DD, *Pdf*^01^, *Pdfr*^*han*5304^, and PDF-ablated flies exhibit a significant increase in total sleep during the subjective day, which is not visible in LD (Chung et al., 2009; Figure 5).

##### Light negatively regulates dopamine and octopamine signalling in l-LNvs

Large ventral-lateral neurons express high levels of dopamine receptors (DopR, DopR2, and D2R) as well as the two major octopamine GPC receptors, OA2 and OAMB (Kula-Eversole et al., 2010). By GRASP (GFP Reconstitution Across Synaptic Partners) analysis (Feinberg et al., 2008) it has been shown that they form membrane contacts with dopaminergic and octopaminergic neurons (Shang et al., 2011). Both dopamine and octopamine represent arousal signals in l-LNvs (Shafer et al., 2008). The response to dopamine is negatively regulated by light and it is time of day-independent, with no significant difference between day/subjective day *versus* the night/subjective night. However, responses in DD are much stronger during both the subjective day and subjective night, in comparison to those at the same circadian times in LD cycles. The effects of octopamine on l-LNvs are both light and time dependent: the responses from subjective day are similar to those of daytime in LD while during subjective night they are far stronger compared to daytime, nighttime, or subjective day (Shang et al., 2011). The time-sensitivity of l-LNvs response to octopamine is a clock-controlled feature, since in *per^01^* mutants the responsiveness during the night is much weaker compared to controls (Shang et al., 2011; Figure 5).

Dopamine signalling in l-LNvs also involves the circadian photoreceptor CRY, expressed at high levels in *Clk*^*Jrk*^ flies, that display a nocturnal behaviour and a reduction in total sleep (Kim et al., 2002; Lu et al., 2008). This CRY-driven nighttime activity of *Clk* mutants is suppressed when dopamine signalling is blocked either pharmacologically or genetically (Kumar et al., 2012).

##### l-LNvs mediate histamine wake-promoting signals

Pigment dispersing factor neurons can receive histaminergic wake-activation signals. Loss-of-function mutations in the *HisCl1* and *hdc* genes result in increased sleep duration, especially during the day (Oh et al., 2013). l-LNvs play an important role in mediating these histaminergic wake-promoting signals: the targeted downregulation of *HisCl1* in PDF cells increases both the daytime and nighttime sleep duration, while the targeted overexpression of *HisCl1* with either *tim*-Gal4 or *Pdf*-Gal4 is able to restore the increased sleep duration of *HisCl1* mutant (Oh et al., 2013; Figure 5).

##### l-LNvs activity is modulated by potassium channels

During sleep, neuronal activity undergoes large-scale changes, and different types of potassium channels are required for normal wake–sleep cycles (Cirelli et al., 2005; Bushey et al., 2007; Allebrandt et al., 2013, 2017). In l-LNvs, the Shal/Kv4, voltage-gated K^+^ channel plays an important role in controlling wake–sleep transition at dusk (Feng et al., 2018). Kv4 acts as sleep-promoter, since flies with a pan-neuronal expression of a dominant-negative form of Kv4 (DNKv4) exhibit a reduced nighttime sleep, as consequence of a decrease in sleep-bout duration. The expression of DNKv4 limited to all PDF positive neurons induces a marked increase in sleep latency and decrease in nighttime sleep, even more evident when the expression is further restricted to l-LNvs (Feng et al., 2018). In l-LNvs, both the frequency of the action potential (AP) currents and the resting membrane potential (RMP) exhibit a strong rhythmicity, with a higher firing rate during daytime and more RMPs significantly depolarized at dawn (ZT1) compared to dusk (ZT13). Both features are dependent on Kv4, since the expression of DNKv4 results in the increase of either the frequency of AP currents or the firing rate during dusk (Feng et al., 2018).

##### l-LNvs and modulation of sleep/wake behaviour at transcriptional level

Many brain neurons, including PDF-positive LNvs, express apterous (ap), a well-known LIM-homeodomain transcription factor involved in development and neuropeptide expression (Hobert and Westphal, 2000; Shimada et al., 2016). ap levels are particularly high in l-LNvs, where they also exhibit a daily oscillation generated by a light-dependent mechanism. In LD both mRNA and protein show a rhythmic expression with a peak during the night (ZT16 and ZT18, respectively), while this oscillation is lost in DD, at least at protein level (Claridge-Chang et al., 2001; Shimada et al., 2016). This transcription factor is involved in buffering light-driven arousal: specific knock-down of *ap* in these PDF neurons results in promoting arousal (reduction in sleep amount and increase in waking time) under LD conditions, whereas the sleep/wake pattern is not affected in DD (Shimada et al., 2016). *ap* knock-down does not significantly affect PDF, neither its expression nor its release; therefore, other neuropeptides or signalling inputs/synaptic output are involved. ap acts in cooperation with the transcription factor Chip (Chi), to drive the expression of developmental genes (Van Meyel et al., 1999). In PDF neurons, the two transcription factors act as a complex playing a key role in transcriptional modulation of sleep/wake behaviour. In fact, while the knock-down of *ap* only results in a general decrease of sleep, regardless of the time of the day, when both proteins are inactive only the daytime sleep amount is decreased. This indicates that this complex modulates mechanisms that act specifically in regulating sleep/wake at different times of the day (Shimada et al., 2016).

#### Small Ventral-Lateral Neurons: A Secondary Role in the Arousal Circuit

##### s-LNvs and PDFR signalling

The arousal activity of l-LNvs is mediated by PDF and a functional PDFR signalling is required for a proper sleep/wake regulation. In fact, *Pdfr* mutants display an increased sleep, specifically during the day (Parisky et al., 2008; Chung et al., 2009; Potdar and Sheeba, 2018; Sheeba et al., 2008a). The PDFR signalling pathway targets the dopaminergic neurons (i.e., PPM3) and plays a crucial role in regulating daytime wakefulness. The downregulation of *Pdfr* in these neurons results in a significant increase of daytime sleep, with longer sleep bouts, while *Pdfr* overexpression suppresses daytime sleep and delays sleep onset (Potdar and Sheeba, 2018).

PDF and dopaminergic neurons are synaptically connected, specifically in the region of s-LNvs axonal projections (Potdar and Sheeba, 2018). Importantly, this observation confirms not only that dopaminergic neurons are a downstream target of PDFR signalling, but also that s-LNvs contribute to the wake-promoting activity of l-LNvs. The involvement of s-LNvs in the arousal circuit was already suggested: (1) the downregulation of *Pdfr* in s-LNvs results in the increase of total sleep (both daytime and nighttime) (Parisky et al., 2008); and (2) the electrical activity of s-LNvs contributes in modulating the phase of evening activity under long photoperiods (Potdar and Sheeba, 2018).

A PDFR signalling originating from the s-LNvs targets also a group of neurons, posterior lateral protocerebrum (PLP) cells, that express the neuropeptide AstA and are involved in sleep promotion (Chen et al., 2016). The thermogenic activation of AstA-PLP neurons causes a significant decrease in locomotor activity and an increase of sleep, either in LD or DD and LL; conversely, the silencing of AstA cells results in a significant reduction of sleep, especially during the midday siesta time, either in LD or DD (Chen et al., 2016).

PLP cells represent downstream target of PDF signalling; they are post-synaptically connected to s-LNvs and express functional PDF receptors and, furthermore, constitutive activation of PDF signalling in AstA-expressing neurons significantly increases the amount of sleep (Chen et al., 2016; Figure 5).

##### s-LNvs and short neuropeptide F (sNPF) signalling

sNPF is broadly expressed in various brain regions, including MB, PI, and CC neurons (Nässel et al., 2008; Johard et al., 2009), and known to regulate different aspects of fly physiology and behaviour (Kahsai et al., 2010; Nagy et al., 2019). sNPF has an important role in promoting and maintaining normal sleep: flies carrying a hypomorphic mutation in *sNPF* or with a knock-down of *sNPF* in adult brain, exhibit a reduced and fragmented sleep compared to control. Moreover, the silencing of sNPF neurons results in a significant reduction of the sleep levels during the daytime (Shang et al., 2013).

The sleep-promoting activity of sNPF neurons is normally suppressed by GABA_*A*_ signalling during the daytime, as the downregulation of the GABA_*A*_ receptor *Rdl* in these neurons leads to a significant increase of both daytime and total sleep time and to a lengthening of sleep bouts (Shang et al., 2013). sNPF is also involved in the response to sleep deprivation: the hyperactivation of sNPF neurons during mechanical sleep deprivation causes a partial sleep-like state and induces less sleep rebound or recovery sleep (Shang et al., 2013).

In s-LNvs sNPF acts in promoting normal nighttime sleep. *sNPF* mRNA levels exhibit a robust oscillation in s-LNvs, while in l-LNvs it is barely expressed (Kula-Eversole et al., 2010). Flies with downregulation of *sNPF* in PDF neurons exhibit a decreases in nighttime sleep, while daytime sleep is not affected (Shang et al., 2013). These sNPF sleep-promoting signals from s-LNvs are transmitted to l-LNvs (Figure 5); the downregulation of *sNPFR* (sNPF receptor) in l-LNvs, where it is normally expressed at high levels (Kula-Eversole et al., 2010), results in a significant fragmentation of nighttime sleep (Shang et al., 2013).

The sleep-promoting role of sNPF is essentially exerted by an inhibitory effect on arousal neurons activity, as it is the result of a balance between the sNPF and the dopamine (DA) signallings in the l-LNvs: in fact, the co-application of DA and sNPF suppresses the cAMP response in the l-LNvs, strongly elicited by DA alone (Shang et al., 2013).

In this section we have focused on those signalling pathways involving PDF neurons specifically related to both light and sleep. Nevertheless, LNvs participate in many other signalling pathways that act in synchronising their activity and regulating sleep/wake behaviour. Among these, (1) glutamatergic transmission mediated by the metabotropic glutamate receptor DmGluRA is important for inhibiting activity in the dark (Hamasaka et al., 2007); (2) GABAergic signalling in the l-LNvs, mediated by GABA_*A*_ receptor Rdl and modulated by the ankyrin repeats domain containing protein WIDE AWAKE (WAKE), plays a role in either the initiation or the maintenance of sleep (Agosto et al., 2008; Parisky et al., 2008; Chung et al., 2009; Liu et al., 2014); and (3) cholinergic inputs to the l-LNvs, mediated by nicotinic acetylcholine receptors (nAChRs) and modulated by glutamate-gated Cl^–^ channels, ensure a highly synchronized rhythmic membrane activity with a simultaneous occurrence of depolarized and hyperpolarized phases (McCarthy et al., 2011).

#### Lateral Posterior Neurons: Connection Between the Clock Network and the Sleep Center

The sleep promoting function of LPNs is modulated by the circadian clock, as the expression of a dominant negative form of *Clock* in these cells reduces the sleep during the daytime (Ni et al., 2019).

The LPN express the neuropeptide AstA and form synaptic connections with the FB; moreover, the inhibition of neurotransmission from the LPNs results in a reduction of sleep (Ni et al., 2019). The excitatory neurotransmitter in LPN that activates FB neurons and promotes sleep is glutamate: in fact, the inhibition of glutamate transport results in a significant reduction of nighttime sleep bouts length and therefore increases sleep fragmentation (Ni et al., 2019).

Fan-shaped body cells are synaptically connected and receive inhibitory input from dopamine arousal (DAA) neurons, as hyperactivation of DAAs antagonizes the effect on sleep promotion observed when LPNs are hyperactivated; moreover, hyperactivation of both LPNs and DAAs significantly fragments sleep (Ni et al., 2019). FB cells promote sleep via GABAergic signalling, as the inhibition of GABA synthesis in these neurons eliminates the sleep promoted by their activation (Ni et al., 2019). FB sleep promoting neurons negatively regulate the activity of OAA neurons: they are closely connected with the FB axon terminals and their neuronal activity is dramatically reduced by the hyperactivation of FB cells (Ni et al., 2019; Figure 3).

#### Dorsal Neurons: A Major Role in Shaping and Maintaining the Sleep/Wake Pattern

Dorsal neurons (DN1s) are at the same time sleep- and wake-promoting cells, as a result of different signalling pathways that either act on different subsets of neurons or are predominant at different times of day, to promote activity in the morning or sleep at siesta and during the night.

A subgroup of DN1 neurons, the posterior DN1s (DN1ps), express the narrow abdomen (na), involved in light-mediated control of diurnal behaviour (morning activity and lights-on response) (Lear et al., 2005). Under LD cycles, DN1ps promote morning activity and their contribution to circadian behaviour is strongly influenced by light intensity (Zhang et al., 2010). They also express PDF receptor that, in these cells, is necessary for periodicity in DD (Zhang et al., 2010).

##### DN1s and DH31 wake-promoting signalling

DN1s secrete the neuropeptide diuretic hormone 31 (DH31) and express its receptor DH31-R1, homologous to vertebrate calcitonin gene-related peptide (CGRP) and its receptor (Mertens et al., 2005). DH31/DH31R are involved in sleep regulation: flies with a loss-of-function mutation in *DH31* exhibit a significant increase of sleep, especially during the night, while the pan-neuronal overexpression of *DH31* significantly decreases nighttime sleep (Kunst et al., 2014). More precisely, DH31 acts as negative regulator of sleep maintenance and awakens flies in anticipation of dawn; indeed the increased sleep of *DH31* mutants is more prominent in the second half of the night and immediately before lights-on, and the overexpressing DH31 flies exhibit a decreased sleep and increase of sleep fragmentation, which is more pronounced in the late night (Kunst et al., 2014). These altered sleep features can be completely rescued by restoring expression of DH31 specifically in DN1s, that can also re-establish the anticipation of the lights-on (Kunst et al., 2014). DN1s are a direct target of PDF signalling from s-LNvs, that modulates sleep by controlling the time of DH31 secretion: PDF specifically activates PDFR late at night, and the consequent secretion of DH31 results in a reduced nighttime sleep and an awakening of flies at dawn (Kunst et al., 2014).

##### DN1s and glutamatergic signalling to clock cells

DN1s neuronal activity is also fundamental in promoting sleep: blocking the synaptic neurotransmission of these cells results in a marked increase of the flies’ activity and a decrease of siesta and nighttime total sleep, due to reduced sleep episodes duration (Guo et al., 2016).

DN1s directly contact core pacemaker cells: GRASP assay identifies a functional direct interaction of DN1s presynaptic regions with either dendritic regions of the Evening cells (the CRY-positive LNds and the 5th s-LNv) or dorsal axon regions of Morning cells (the s-LNvs) (Guo et al., 2016). This neuronal transmission is mediated by glutamatergic signalling, with inhibitory effect. DN1s express the vesicular glutamate transporter, DvGluT, while E cells express the metabotropic glutamate receptor DmGluRA, whose mRNA exhibits cycling levels with a peak in the middle of the day. This support the predominant inhibitory role of DN1s on E cell-derived locomotor activity, which is then confined in the late day-early night (Guo et al., 2016).

##### DN1s signals to sleep center

DN1s can be both wake- and sleep- promoting, according to synaptic types and targets. A CRY-positive subset of DN1s (anterior-projecting DNs, APDNs or a-DN1ps) sends post-synaptic projections also to the anterior region of adult brain, the superior lateral protocerebrum, target of the AstA sleep-promoting signalling from PLPs (Guo et al., 2016), and innervates the anterior optic tubercle (AOTU) (Guo et al., 2018; Lamaze et al., 2018). In particular, they target a small subset of neurons within the AOTU (TuBu), that receive visual inputs from medullo-tubercular neurons and transmit this information to EB-R neurons (Guo et al., 2018; Lamaze et al., 2018). However, DN1ps connect TuBu using both excitatory and inhibitory synapses. They suppress the sleep-promoting activity of TuBu neurons, as their acute inhibition results in an increase of TuBu electrical activity, while thermo-genetic excitation of TuBu neurons profoundly induces sleep throughout both day and night (Lamaze et al., 2018). On the other hand, DN1ps activate TuBu using glutamate, giving sleep-promoting effect (Guo et al., 2018).

Moreover, a CRY-negative subset of DN1s (ventro-contralateral-projecting DN1p neurons, vc-DN1ps) send ventral and contralateral projections to the PI region (Cavanaugh et al., 2014), that represents an activity-promoting output of DN1s, since the activation of these cells promotes wakefulness and inhibits sleep (Guo et al., 2018).

## Light Exposure: Timing and Intensity

The quality and architecture of sleep is also influenced by the characteristics of the light stimulus, such as the intensity of light and the timing of light exposure (morning/daytime versus evening/nighttime).

### Nocturnal Light Affects Daytime Sleep

Natural pattern of light availability assumes dark nighttime, therefore a different administration of light disrupts the sleep pattern. Sleep analysis of flies exposed to 4 days of discontinuous nocturnal light stimulation (DLS) showed a reduction of sleep episode duration (but not the sleep bouts number) specifically during the day, while it does not affect nighttime sleep. Moreover, during recovery time after light disruption, the quality of nighttime sleep is increased, opposite to daytime sleep. On the molecular level, discontinuous light stimulation disrupts CRY daily oscillation at both mRNA and protein levels, and decreases TIM levels during the night (Liu and Zhao, 2014).

### Light Intensity Impacts on Sleep Timing

In the natural environment, light intensity changes between 0 and 100,000 lux, depending on the time of the day, time of the year, weather, etc. Insects are sensitive to a broad range of light, and they can adjust their behaviour according to light exposure, through an adaptative mechanism that allows avoiding of bright light in the middle of the day, especially during summertime (Lazopulo et al., 2019).

Flies are able to detect low light intensity by using CRY (Vinayak et al., 2013) and the four rhodopsins expressed in photoreceptors cells in the compound eyes (Rh1, Rh3, Rh4, Rh6) (Saint-Charles et al., 2016). In natural conditions, dim light appears during full moon nights and around dawn and dusk. Moonlight causes the phase shift of molecular clock in pacemaker cells, resulting in the advance of morning activity and the delay of the evening one, with the overall flies’ activity becoming more nocturnal. Experiments performed with clock mutants have clearly demonstrated that the effect on nighttime activity is light-dependent (Kempinger et al., 2009) and the response to moonlight is mediated by R1–6 and Rh6-expressing R8 photoreceptors (Schlichting et al., 2014), while CRY is not involved (Bachleitner et al., 2007). Moreover, dim light does not affect clock protein expression pattern in peripheral oscillators in the retina (Bachleitner et al., 2007). Periodogram analysis of wt flies under L:ML conditions (Light:MoonLight) reveals that sleep time is also affected: relative level of activity is increased compared with flies reared in light:dark conditions, and activity is continuous during the whole night, with flies sleeping mostly during the day (Schlichting et al., 2014).

The effect of twilight is opposite to moonlight: morning peak of activity is delayed and evening peak is advanced, while nocturnal activity in reduced (Rieger et al., 2007). R7 and R8 photoreceptors are involved in the response to twilight (Schlichting et al., 2014). When flies are exposed to both dim light at dawn/dusk and moonlight, the twilight effect dominates in terms of shifted morning and evening peak of activity and reduced nocturnal locomotor activity. This more composite light exposure has an effect which is more similar to natural conditions: fly activity is not shifted to the nighttime during full moon nights (Schlichting et al., 2014; Vanin et al., 2012).

In the middle of the day, flies are exposed to high intensity light (HI). Response to HI is independent from CRY, ocelli, and compound eyes, and most probably it is mediated by HB eyelets (Schlichting et al., 2016), which communicate with s-LNvs through acetylcholine. The possible mechanism at the basis of activity and sleep regulation mediated by HI assumes that activation of acetylcholine receptor on s-LNvs increases Ca^2+^ level, which, in turn, causes delayed PER degradation during the day. Then, s-LNvs propagate signal through PDF pathway, affecting PER cycling in downstream neurons DN1s, known to be sleep regulators (Guo et al., 2016). Indeed, flies exposed to HI show delayed evening peak of activity and lengthened siesta time (Schlichting et al., 2019a), thus avoiding bright light in the middle of the day during hot summer time.

### Light Influences Temperature-Dependent Regulation of Sleep

Light plays a role in the regulation of temperature-dependent sleep pattern. Nighttime sleep is decreased by high temperature, but this effect is influenced by light presence during the preceding day, as it was shown in experiments performed in DD conditions. The mechanism of this process requires CRY, as *cry*^*out*^ mutants do not show decreased nighttime sleep in response to heat (Parisky et al., 2016). This effect seems to be connected with the wake-promoting role of dopamine on nighttime sleep, as it was shown that light increases expression of inhibitory dopamine receptors (Shang et al., 2011). The involvement of light in temperature-dependent sleep control was comprehensively reviewed elsewhere (Lamaze and Stanewsky, 2020).

## Conclusion

Understanding the mechanisms underlying the relations between light exposure and sleep disturbances has become a challenge. Modern society no longer relies on the day-night differences in external conditions that have shaped life on Earth: from shift-work schedules to the current widespread use of digital technology until late at night, we are more and more exposed to stimuli that not only are not coordinated with our body’s internal time but can also stimulate alertness and extend sleep latency. Taking advantage of the fruit fly *Drosophila melanogaster*, we have tried to address the contribution of the different light signalling pathways involved in promoting, consolidating, or preventing sleep. The picture derived is complex: the architecture of sleep is regulated by an intricate set of structures, neurotransmitters, and networks that integrate environmental signals.

However, as most of the essential sleep features are shared between flies and mammals, the knowledge of how light regulates this complex behaviour in *Drosophila* can be fundamental for future research in humans, addressing how light can promote high wakefulness during the day and good sleep during the night.

## Author Contributions

GM, MD, and PC equally contributed to writing the manuscript. All authors contributed to the article and approved the submitted version.

## Conflict of Interest

The authors declare that the research was conducted in the absence of any commercial or financial relationships that could be construed as a potential conflict of interest.
